# A case report of IgA-mediated anti-Laminin-γ1 (p200) pemphigoid successfully treated with stapokibart combined with corticosteroids

**DOI:** 10.3389/fimmu.2026.1767277

**Published:** 2026-01-27

**Authors:** Ting Zhang, Yanhong Liu, Zhaohuai Zhang, Ying Wang, Zhimin Lin, Zhenhua Nie, Chen Li, Zuotao Zhao

**Affiliations:** 1 Department of Dermatology, Tianjin Institute of Integrative Dermatology, Tianjin Academy of Traditional Chinese Medicine Affiliated Hospital, Tianjin, China; 2 Beijing University of Chinese Medicine, Beijing, China; 3 Department of Immunology, School of Basic Medical Sciences, the National Health Commission Key Laboratory of Medical Immunology, Medicine Innovation Center for Fundamental Research on Major Immunology-related Diseases, Peking University, Beijing, China; 4 Center for Human Disease Genomics, Peking University, Beijing, China; 5 School of Clinical Medicine, Beijing University of Chinese Medicine, Beijing, China; 6 Department of Dermatology, Shenzhen People’s Hospital, Guangdong, Shenzhen, China

**Keywords:** anti-Laminin-γ1 pemphigoid, autoimmune, case report, facial erosions, palmoplantar blisters

## Abstract

IgA-mediated Anti-Laminin-γ1 (p200) pemphigoid is a rare subtype of subepidermal autoimmune blistering disease (AIBD) characterized by IgA autoantibodies targeting the laminin γ1 chain, a 200 kDa protein located at the dermal-epidermal junction. Patients typically exhibit skin-dominant blistering lesions, also mucosal involvement has been reported. We report a 73-year-old male patient diagnosed with IgA-mediated anti-Laminin-γ1 (p200) pemphigoid. He initially presented with prominent palmoplantar blisters erosions on the eyelids, lips and face, accompanied by mild pruritus. Skin biopsy demonstrated hyperkeratosis, parakeratosis, acanthosis, subepidermal blister formation, and mild perivascular lymphocytic infiltration in the superficial dermis. Direct immunofluorescence showed C3 deposits on the basement membrane. The patient was initially treated successfully with methylprednisolone. During the second recurrence, he presented with annular erythema topped with blisters and erosions on the lips. Comprehensive pemphigus and pemphigoid antibodies panel test showed Laminin γ1 IgA with a titer of 1:10. The rash recurred when corticosteroids were tapered. The patient was then treated with Stapokibart (CM310) in combination with corticosteroids, leading to complete resolution of the skin lesions and successful tapering of the corticosteroids. This case represents the first successful treatment of IgA-mediated anti-Laminin-γ1 (p200) pemphigoid using Stapokibart in combination with corticosteroid.

## Introduction

IgA-mediated anti-p200 pemphigoid is a rare variant of subepidermal autoimmune bullous disease (AIBD) characterized by circulating IgA autoantibodies targeting the p200 antigen (a 200 kDa protein at the basement membrane zone). which distinct from IgG-mediated anti-p200 pemphigoid. It was first reported by Wozniak K in 2011 (1) and reported the second case in 2025 (2). Immunoelectron microscopy studies revealed that the epitopes recognized by IgA antibodies are located below the lamina densa, similar to the localization of IgG antibodies, a distinct epitope recognition pattern compared to other subtypes (such as IgA-MMP or IgA-EBA), suggesting it may represent a distinct clinical entity (2). Clinically, it may resemble bullous pemphigoid, linear IgA bullous dermatosis, or dermatitis herpetiformis (3). In mucosal pemphigoid (MMP), the presence of IgA anti-laminin γ1 antibodies has also been observed. A unique case demonstrated IgA reactivity on the dermal side by indirect immunofluorescence (salt-split skin), and immunoblotting confirmed the presence of IgA autoantibodies targeting LMγ1 (4).

Since IgA-mediated anti-p200 pemphigoid is rare reported, treatment strategies are mostly extrapolated from Linear IgA bullous dermatosis (LABD), Dapsone is the most commonly recommended treatment for LABD, as it is often effective in controlling the disease (5). When dapsone is not accessible or not tolerated, other treatment may include, such as systemic corticosteroids, immunosuppressants, biological agents which are mentioned for autoimmune bullous diseases in general (6). The 2 cases which have been reported before, they both were treated with dapsone combined with corticosteroids. Similar to Dupilumab, New biologics Stapokibart which targets the interleukin-4 receptor alpha subunit (IL-4Rα) effectively blocks the signaling of interleukin-4 (IL-4) and interleukin-13 (IL-13), demonstrated clinical efficacy in treating moderate-to-severe atopic dermatitis (AD) (7), chronic rhinosinusitis with nasal polyps (8) and seasonal allergic rhinitis in clinic trials (9). However, It has not been reported for the treatment of pemphigoid or Linear IgA bullous dermatosis (LABD).

Here, we report a 73-year-old male patient diagnosed with IgA-mediated anti-Laminin-γ1 (p200) pemphigoid. To our knowledge, this is the third reported case of IgA-mediated Anti-Laminin-γ1 (p200) pemphigoid. The two cases reported previously received dapsone combined with corticosteroids. This patient was initially treated successfully with methylprednisolone. However, two relapses occurred during steroid tapering. The addition of Stapokibart ultimately enabled successful steroid reduction and maintenance. This case represents the first successful treatment of IgA-mediated anti-Laminin-γ1 (p200) pemphigoid using Stapokibart in combination with corticosteroids.

## Case report

**Figure 1:**
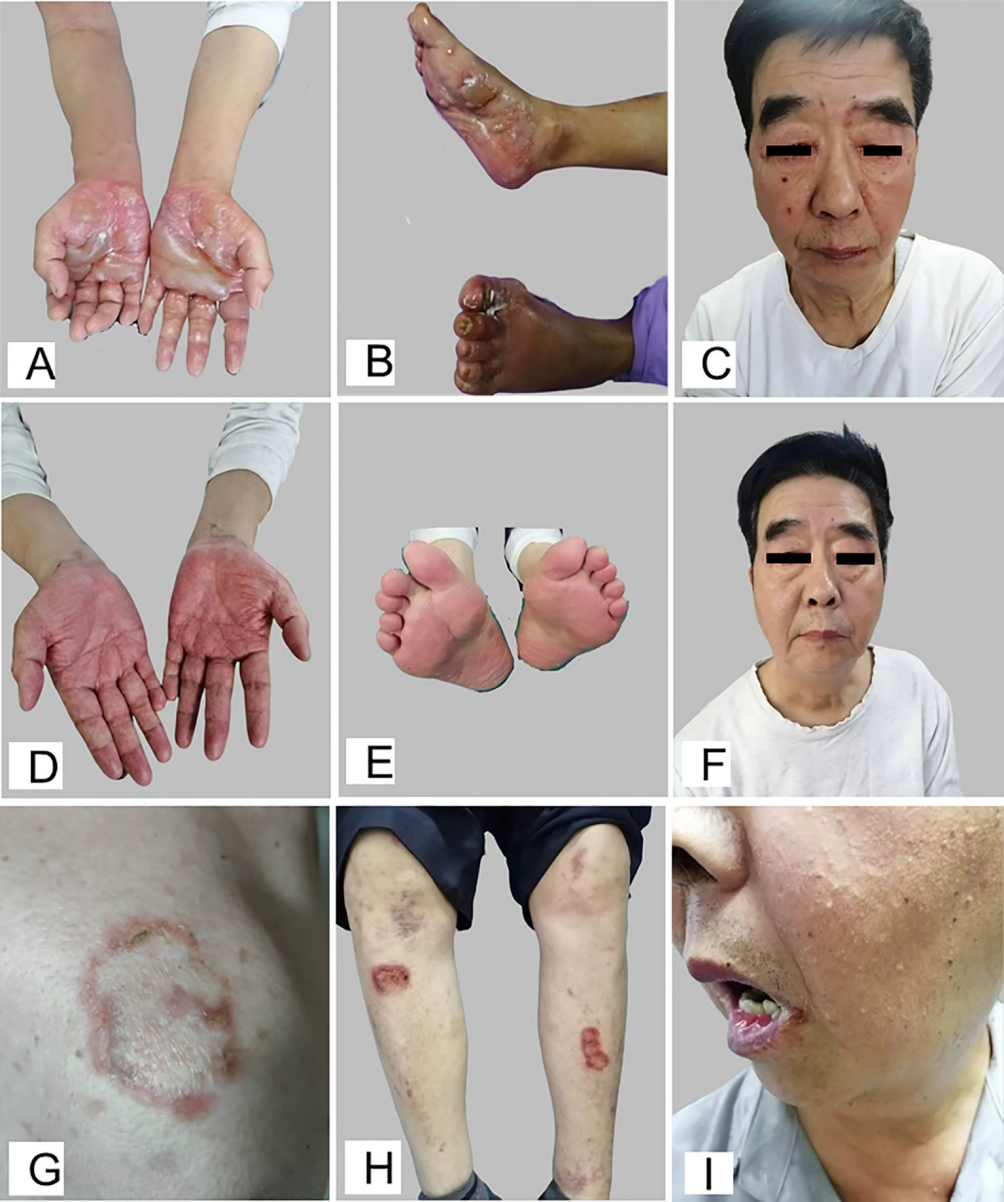
Multiple vesicles and bullae, partially coalescing into larger bullae, were present on the palms **(A)** and soles **(B)**. Erosions were also observed on the eyelids, lips, and facial skin **(C)**. Following treatment, the blisters on the palms **(D)** and soles **(E)**, along with the facial erosions **(F)**, resolved completely. featuring annular erythema on the right shoulder **(G)** and anterior lower leg surmounted by multiple blisters **(H)**, accompanied by erosions on the lips **(I)**.

On March 22, 2025, a 73-year-old male presented to our dermatology clinic with a 6-day history of bullous lesions on the palms and soles and erosive lesions on the face. The initial manifestations consisted of erosions on the lips, along with erythematous patches and blisters on the palms and soles. The condition subsequently progressed, with the appearance of multiple erosions on the eyelids and face (without evident blister formation in these areas), a progressive increase in blisters on the palms and soles, and the emergence of new, scattered vesicles on the upper arms and lower abdomen. The patient reported mild pruritus. There was no history of suspicious medication use or infection prior to symptom onset. His medical history included generalized pruritus for two years without visible rash and longstanding darkened urine, both of which had not been medically investigated. He also had a 10-year history of hypertension, which was well-controlled with oral irbesartan. Dermatological examination revealed multiple tense blisters on the palms and soles, some of which had coalesced into larger bullae, in addition to scattered blisters on the upper arms and lower abdomen. Erosions were observed on the eyelids, lips, and face, again without visible blisters in these areas. The oral mucosa was unaffected. The findings are illustrated in **Figures 1A–C**. Based on the clinical presentation, the differential diagnoses included pemphigoid and erythema multiforme. The patient was hospitalized for further investigation and management.

**Figure 2:**
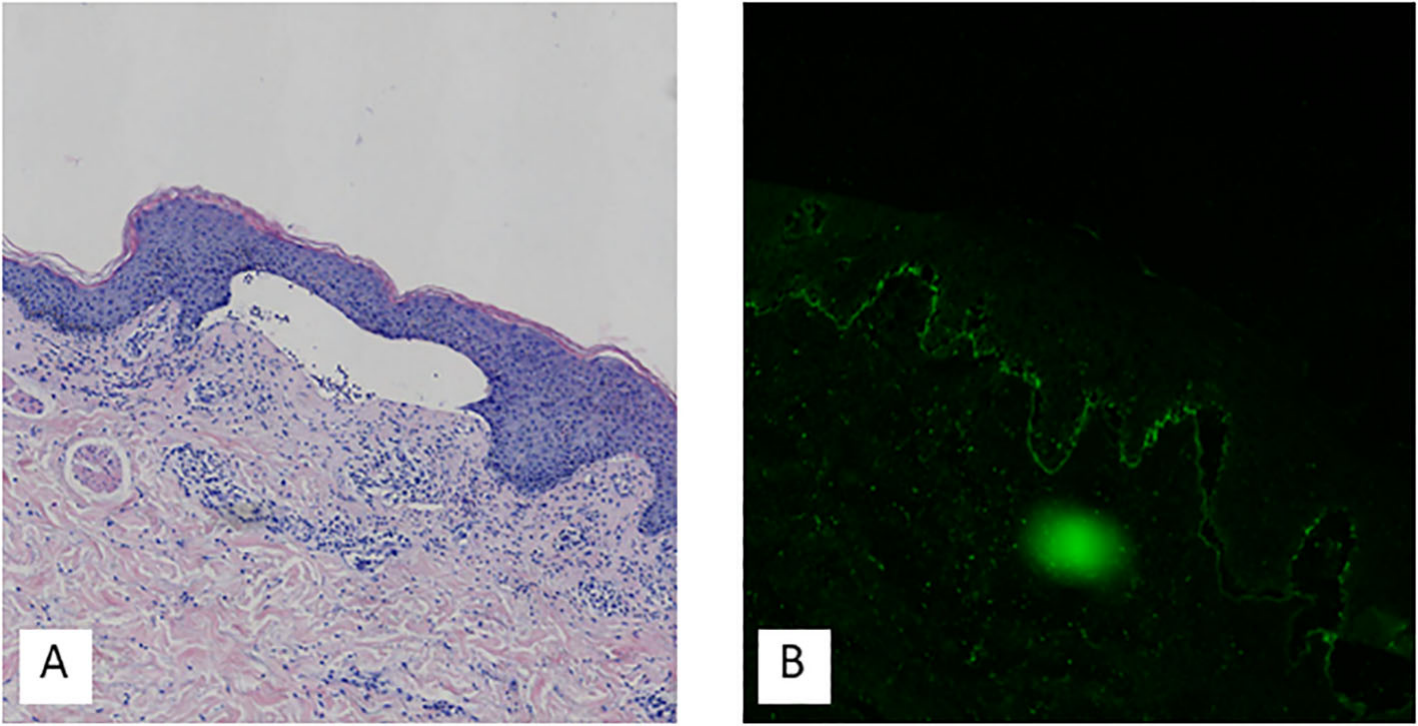
Hematoxylin-eosin staining showed hyperkeratosis, parakeratosis, acanthosis, subepidermal blister formation, and mild perivascular lymphocytic infiltration in the superficial dermis **(A)**. Direct immunofluorescence demonstrated C3 deposits on the basement membrane **(B)**.

A skin biopsy was obtained from the right upper arm. Histopathological examination with hematoxylin and eosin (H&E) staining revealed hyperkeratosis, parakeratosis, acanthosis, subepidermal blister formation, and a mild perivascular lymphocytic infiltrate in the superficial dermis. Direct immunofluorescence (DIF) demonstrated linear deposits of C3 along the basement membrane zone, as shown in **Figure 2**. Serological testing for circulating autoantibodies, including BP180, BP230, Dsg1, and Dsg3, returned negative results. A comprehensive immunological profile revealed no significant abnormalities. However, hematological investigations showed neutrophilic leukocytosis, along with elevated levels of inflammatory markers, including C-reactive protein (CRP), serum amyloid A (SAA), erythrocyte sedimentation rate (ESR), and D-dimer. Laboratory tests also indicated a marked increase in total IgE, and urinalysis confirmed hematuria. Procalcitonin (PCT) levels and liver and kidney function tests were within normal limits. Serological tests for hepatitis B surface antigen, hepatitis C antibody, syphilis, and HIV were negative. Tumor markers, including AFP, CEA, CA724, and CA199, were not elevated. Imaging studies included a chest CT scan, which identified right middle lobe bronchial obstruction with associated atelectasis, localized emphysema in both lungs, and arteriosclerosis. Abdominal ultrasonography demonstrated mild fatty liver and multiple hepatic cysts. Renal ultrasonography revealed a left renal cyst and prostatic hyperplasia with calcification. Based on the clinical presentation and supportive histopathological and immunofluorescence findings, a diagnosis of pemphigoid was established.

The patient was initiated on intravenous methylprednisolone 100 mg/day, supplemented with topical halometasone and daily drainage of bullous lesions. After five days of treatment, laboratory tests showed a marked decrease in inflammatory markers, including CRP, SAA, ESR, and D-dimer, and urinalysis indicated resolution of hematuria. Significant clinical improvement of the skin lesions was observed by day 10. By hospital day 17, the corticosteroid was tapered and switched to oral triamcinolone at 48 mg/day. The patient was discharged following complete resolution of the rash, as shown in **Figures 1D–F**.

During outpatient follow-up, the triamcinolone dose was gradually tapered. However, three months after discharge (July 10, 2025), when the dose was reduced to 16 mg/day, the disease recurred with the appearance of new blisters on the palms and soles. According to medical orders, the triamcinolone dose was subsequently increased to 48 mg/day. This intervention led to substantial resolution of the skin lesions within two weeks. On August 16, 2025, during a corticosteroid taper at a dose of triamcinolone 28 mg/day, the patient experienced a recurrence. This episode presented with a distinct morphology, featuring annular erythema on the right shoulder and anterior lower leg surmounted by multiple blisters, accompanied by erosions on the lips, as shown in **Figures 1G–I**. A comprehensive pemphigus and pemphigoid antibody panel was subsequently performed (**Table 1**). The results were positive for Laminin γ1 IgA at a titer of 1:10, confirming a diagnosis of IgA related Laminin γ1 (p200) pemphigoid.

**Table 1:** Serum pemphigus and pemphigoidantibody test.

Pemphigus and pemphigoid antibodies	Test method	result	Reference value
Desmoglein1(Dsg1)antibody IgG	Cell based assay (CBA)	Negative (-)	Negative (-)
Desmoglein 1(Dsg1)antibody IgA	Cell based assay (CBA)	Negative (-)	Negative (-)
Desmoglein 3(Dsg3)antibody IgG	Cell based assay (CBA)	Negative (-)	Negative (-)
Desmoglein 3(Dsg3)antibody IgA	Cell based assay (CBA)	Negative (-)	Negative (-)
BP180 antibody IgG	Cell based assay (CBA)	Negative (-)	Negative (-)
BP180 antibody IgA	Cell based assay (CBA)	Negative (-)	Negative (-)
BP230 antibody IgG	Cell based assay (CBA)	Negative (-)	Negative (-)
BP230 antibody IgA	Cell based assay (CBA)	Negative (-)	Negative (-)
Anti-Type VII Collagen Antibodies IgG	Cell based assay (CBA)	Negative (-)	Negative (-)
Anti-Type VII Collagen Antibodies IgA	Cell based assay (CBA)	Negative (-)	Negative (-)
Anti-Laminin-332 Antibodies IgG	Cell based assay (CBA)	Negative (-)	Negative (-)
Anti-Laminin-332 Antibodies IgA	Cell based assay (CBA)	Negative (-)	Negative (-)
Anti-Laminin-γ1 (p200) Antibodies IgG	Cell based assay (CBA)	Negative (-)	Negative (-)
Anti-Laminin-γ1 (p200) Antibodies IgA	Cell based assay (CBA)	Positive (+) 1:10	Negative (-)
Anti-Integrin α6 Antibodies IgG	Cell based assay (CBA)	Negative (-)	Negative (-)
Anti-Integrin α6 Antibodies IgA	Cell based assay (CBA)	Negative (-)	Negative (-)
Anti-Integrin β4 Antibodies IgG	Cell based assay (CBA)	Negative (-)	Negative (-)
Anti-Integrin β4 Antibodies IgA	Cell based assay (CBA)	Negative (-)	Negative (-)

Given that long-term corticosteroid therapy had already induced complications including hyperglycemia and coronary heart disease, the medical team opted not to escalate the steroid dose. Instead, Tripterygium wilfordii (Thunder God Vine) was introduced at 20 mg three times daily. The skin lesions gradually subsided over the following two weeks. However, on September 19, 2025, as the triamcinolone dose was reduced to 20 mg/day, new lesions emerged. The treatment regimen was therefore escalated to include Stapokibart, administered subcutaneously with a 600 mg loading dose, followed by a maintenance dose of 300 mg every two weeks. This intervention led to substantial resolution of the rash by September 30. Subsequent tapering was accomplished without recurrence: the Tripterygium wilfordii dose was reduced to 10 mg three times daily on October 16, 2025, and the triamcinolone was tapered to 16 mg/day by November 1, 2025. The patient remains on maintenance therapy with Stapokibart 300 mg subcutaneously every two weeks and has exhibited no recurrence of skin lesions to date.

## Discussion

IgA-mediated bullous diseases constitute a subset of autoimmune blistering disorders characterized by IgA autoantibodies targeting structural proteins in the skin and mucous membranes. This group primarily includes linear IgA bullous dermatosis (LABD) and IgA pemphigus. LABD is defined by linear deposition of IgA at the with tense blisters, erosions, and mucosal involvement, which may resemble bullous pemphigoid (BP) in certain cases (5, 10). IgA pemphigus, a rare variant, is mediated by IgA autoantibodies against desmogleins (e.g., desmoglein 3), resulting in intraepidermal acantholysis. Its pathogenesis involves Fcα receptor (FcαRI)-mediated neutrophil activation (11). First-line treatment for LABD typically includes dapsone and corticosteroids. However, dapsone carries risks of hematologic toxicity, while corticosteroids may induce metabolic disorders, osteoporosis and other adverse effects. In refractory cases, conventional immunosuppressants may be considered, though they are not always effective and are associated with significant side effects. Biologics such as rituximab (anti-CD20), omalizumab (anti-IgE), and dupilumab (anti-IL-4Rα) have shown promise in treating IgG-mediated bullous pemphigoid (12). Nevertheless, targeted therapies for IgA-mediated diseases remain underdeveloped (5). To date, the use of anti-IL-4Rα biologics such as dupilumab or stapokibart has not been reported in the treatment of IgA-mediated bullous diseases. The potential application of these agents may offer clinicians new therapeutic options in the future.

Anti-Laminin-γ1 (p200) pemphigoid is a rare subepidermal autoimmune blistering disease (AIBD) characterized by autoantibodies targeting a 200 kDa protein, with laminin γ1 identified as the primary antigen in 70%-90% of cases (13). Despite its recognition, the pathogenic role of anti-laminin γ1 autoantibodies remains unclear. Recent studies have identified laminin β4 as an additional target antigen in anti-p200 pemphigoid, which localizes at the basement membrane zone and is predominantly expressed in keratinocytes, inducing dermal-epidermal separation via leukocyte activation and reactive oxygen species (ROS) release (14).

IgA-mediated Anti-Laminin-γ1 (p200) pemphigoid is a unique subset of this disease involves IgA-mediated autoimmunity against laminin γ1, as evidenced by cases of mucous membrane pemphigoid (MMP) with IgA reactivity to laminin γ1 in immunoblotting and indirect immunofluorescence (4). This IgA variant underscores the heterogeneity of anti-p200 pemphigoid, Clinically, it may resemble bullous pemphigoid, linear IgA bullous dermatosis, or dermatitis herpetiformis, which may present with mucosal lesions, though the mechanism linking IgA autoantibodies to mucosal pathology is poorly understood (4).

IgA-mediated anti-laminin-γ1 (p200) pemphigoid can be diagnosed using a combination of specialized laboratory techniques. Immunoprecipitation and immunoblotting are employed to identify laminin γ1 as the target antigen in patient sera. In these assays, skin extracts and patient sera are analyzed to detect the presence of autoantibodies directed against laminin γ1 (15). Indirect immunofluorescence (IF) is another key method used to detect IgA autoantibodies bound to laminin γ1 within the basement membrane zone (BMZ) of normal human skin or salt-split skin substrates. A positive IgA signal localized to the dermal side of salt-split skin is strongly suggestive of the diagnosis (16). Mass spectrometry is utilized for precise antigen identification and for characterizing the molecular interactions between laminin γ1 and other laminin chains (e.g., α3, γ2) (17). Immunoelectron microscopy provides high-resolution localization of the epitopes recognized by IgA autoantibodies within the BMZ, offering valuable structural insights, although it is a relatively time-consuming technique (2). The differentiation of anti-p200 pemphigoid from other AIBDs, such as laminin-332 pemphigoid or bullous pemphigoid, relies on identifying specific autoantigens (e.g., laminin β4 or γ1) (2). Due to limitations in the hospital laboratory conditions, serum anti laminin β4 was not tested for this patient.

Currently, there is a lack of large-scale clinical trial data regarding the treatment of IgA-mediated anti-p200 pemphigoid. Consequently, therapeutic approaches are primarily extrapolated from those used for linear IgA bullous dermatosis (LABD). Dapsone is considered a first-line treatment, with an initial dose typically starting at 0.5 mg/kg/day. The dosage may be titrated upward based on clinical response and patient tolerability (18). Treatment is continued until clinical remission is achieved, often followed by maintenance therapy to prevent relapse (5). Systemic corticosteroids are frequently used in combination with dapsone or other immunomodulatory agents, such as doxycycline, and are administered at variable doses. The corticosteroid regimen is gradually tapered in accordance with clinical response (18). In severe or refractory cases, immunosuppressants such as azathioprine or mycophenolate mofetil may be considered (19, 20). Intravenous immunoglobulin (IVIg) has also shown potential efficacy in refractory disease Recent research has explored targeted therapeutic strategies, including anti-CD89 monoclonal antibodies, which inhibit IgA-induced neutrophil activation and have demonstrated promise in preclinical LABD models (21). Similarly, anti-FcαRI monoclonal antibodies have been shown to prevent disease onset and resolve established inflammation in experimental settings. New biologics Stapokibart is a humanized IgG4 monoclonal antibody that specifically targets the interleukin-4 receptor alpha subunit (IL-4Rα). It shares a similar mechanism of action with Dupilumab. By binding to IL-4Rα, it effectively blocks the signaling of two key type 2 cytokines: interleukin-4 (IL-4) and interleukin-13 (IL-13), which play crucial roles in the pathogenesis of various type 2 inflammatory diseases (22). It has demonstrated clinical efficacy in treating moderate-to-severe atopic dermatitis (AD) (7). Beyond AD, stapokibart has shown promise in other type 2 inflammatory conditions: such as chronic rhinosinusitis with nasal polyps (8), seasonal allergic rhinitis (9). Stapokibart received its first approval in China (September 2024) for moderate-to-severe AD, followed by approvals for chronic rhinosinusitis with nasal polyps (December 2024) and seasonal allergic rhinitis (February 2025) (22). It represents a novel therapeutic option for multiple type 2 inflammatory diseases by targeting the shared IL-4/IL-13 pathway. Mild to moderate adverse events were frequently reported, with 68.0% of patients experiencing them in one study (23). The most common treatment-emergent adverse events (TEAEs) included upper respiratory tract infections, conjunctivitis, cough, and dyspnea. Injection site reactions were mostly mild (24). Serious adverse events were rare, occurring in 2.2% of Stapokibart-treated patients compared to 1.1% in the placebo group. Arthralgia was reported in 7.8% of patients receiving Stapokibart versus 0% in the placebo group. Hyperuricemia was observed in 5.6% of treated patients compared to 1.1% in the placebo group (8). Some studies also noted occurrences of sinus bradycardia and hyperlipidemia (25). No new safety signals were identified in long-term studies lasting up to 52 weeks (7). When combined with corticosteroids, no significant additional risks were reported, though larger controlled studies are needed for confirmation (25). To date, there have been no reported treatments for IgA-mediated bullous diseases using this agent. The present case represents the first successful treatment of IgA-mediated anti-Laminin-γ1 (p200) pemphigoid with Stapokibart in combination with corticosteroids. The patient has shown no recurrence of skin lesions, and no significant side effects have been observed to date.

## Data Availability

The original contributions presented in the study are included in the article/supplementary material. Further inquiries can be directed to the corresponding authors.

## References

[1] Wozniak K Hashimoto T Fukuda S Ohyama B Ishii N Koga H . IgA anti-p200 pemphigoid. Arch Dermatol. (2011) 147:1306–10. doi: 10.1001/archdermatol.2011.303, PMID: 22106117

[2] Kowalewski C Wozniak K . Linear IgA bullous dermatosis-a fifty year experience of Warsaw Center of bullous diseases. Front Immunol. (2024) 15:1478318. doi: 10.3389/fimmu.2024.1478318, PMID: 39877369 PMC11772161

[3] Gao Y Qian H Hashimoto T Li X . Potential contribution of anti-p200 autoantibodies to mucosal lesions in anti-p200 pemphigoid. Front Immunol. (2023) 14:1118846. doi: 10.3389/fimmu.2023.1118846, PMID: 36761755 PMC9905711

[4] Kuang W Qian H Zhang Q Li W Hashimoto T Zeng X . Case Report: Mucous Membrane Pemphigoid With IgG and IgA Anti-Laminin gamma1 Antibodies and IgA Anti-Laminin alpha5 Antibodies. Front Immunol. (2022) 13:903174. doi: 10.3389/fimmu.2022.903174, PMID: 35720393 PMC9198329

[5] Mar K Landells F Khalid B Sriranganathan A Wang OJ Starkey SY . Clinical characteristics and treatment outcomes of linear IgA bullous dermatosis. J Dtsch Dermatol Ges. (2025) 23:576–85. doi: 10.1111/ddg.15644, PMID: 40018881 PMC12087753

[6] Huang D Zhang Y Kong L Lu J Shi Y . Janus kinase inhibitors in autoimmune bullous diseases. Front Immunol. (2023) 14:1220887. doi: 10.3389/fimmu.2023.1220887, PMID: 37492565 PMC10363722

[7] Zhao Y Zhang L Wu L Yang B Wang J Li Y . Long-term efficacy and safety of stapokibart for moderate-to-severe atopic dermatitis: 52-week results from a phase 3 trial. Allergy. (2025) 80:1348–57. doi: 10.1111/all.16368, PMID: 39450683

[8] Shen S Yan B Wang M Wu D Piao Y Tang J . Stapokibart for severe uncontrolled chronic rhinosinusitis with nasal polyps: the CROWNS-2 randomized clinical trial. Jama. (2025) 334:962–72. doi: 10.1001/jama.2025.12515, PMID: 40824573 PMC12362275

[9] Zhang Y Li J Wang M Li X Yan B Liu J . Stapokibart for moderate-to-severe seasonal allergic rhinitis: a randomized phase 3 trial. Nat Med. (2025) 31:2213–21. doi: 10.1038/s41591-025-03651-5, PMID: 40186079 PMC12283386

[10] Zhou Y Zhou X Feng X Xia D Qian H Liu H . Case Report: Prurigo nodularis-like linear IgA/IgG bullous dermatosis: a case report and literature review. Front Immunol. (2023) 14:1201163. doi: 10.3389/fimmu.2023.1201163, PMID: 37325615 PMC10265503

[11] Emtenani S Ghorbanalipoor S Mayer-Hain S Kridin K Komorowski L Probst C . Pathogenic activation and therapeutic blockage of fcαR-expressing polymorphonuclear leukocytes in IgA pemphigus. J Invest Dermatol. (2021) 141:2820–8. doi: 10.1016/j.jid.2021.06.007, PMID: 34246620

[12] Cao P Xu W Zhang L . Rituximab, omalizumab, and dupilumab treatment outcomes in bullous pemphigoid: A systematic review. Front Immunol. (2022) 13:928621. doi: 10.3389/fimmu.2022.928621, PMID: 35769474 PMC9235912

[13] Goletz S Pigors M Lari TR Hammers CM Wang Y Emtenani S . Laminin beta4 is a constituent of the cutaneous basement membrane zone and additional autoantigen of anti-p200 pemphigoid. J Am Acad Dermatol. (2024) 90:790–7. doi: 10.1016/j.jaad.2023.11.014, PMID: 37992812

[14] Pigors M Goletz S Wang Y Emtenani S Hammers CM Holtsche MM . Anti-Laminin beta4 IgG Drives Tissue Damage in Anti-p200 Pemphigoid and Shows Interactions with Laminin alpha3 and gamma1/2 Chains. J Invest Dermatol. (2025) 145:821–30.e3. doi: 10.1016/j.jid.2024.08.004, PMID: 39320300

[15] Goletz S Pigors M Lari TR Hammers CM Wang Y Emtenani S . Laminin β4 is a constituent of the cutaneous basement membrane zone and additional autoantigen of anti-p200 pemphigoid. J Am Acad Dermatol. (2024) 90:790–7. doi: 10.1016/j.jaad.2023.11.014, PMID: 37992812

[16] Kuang W Qian H Zhang Q Li W Hashimoto T Zeng X . Case report: mucous membrane pemphigoid with IgG and IgA Anti-Laminin γ1 antibodies and IgA Anti-Laminin α5 antibodies. Front Immunol. (2022) 13:903174. doi: 10.3389/fimmu.2022.903174, PMID: 35720393 PMC9198329

[17] Pigors M Goletz S Wang Y Emtenani S Hammers CM Holtsche MM . Anti-Laminin β4 IgG drives tissue damage in anti-p200 pemphigoid and shows interactions with laminin α3 and γ1/2 chains. J Invest Dermatol. (2025) 145:821–30.e3. doi: 10.1016/j.jid.2024.08.004, PMID: 39320300

[18] Beek NV Zillikens D Schmidt E . Bullous autoimmune dermatoses–clinical features, diagnostic evaluation, and treatment options. Dtsch Arztebl Int. (2021) 118:413–20. doi: 10.3238/arztebl.m2021.0136, PMID: 34369370 PMC8380840

[19] Montagnon CM Lehman JS Murrell DF Camilleri MJ Tolkachjov SN . Subepithelial autoimmune bullous dermatoses disease activity assessment and therapy. J Am Acad Dermatol. (2021) 85:18–27. doi: 10.1016/j.jaad.2020.05.161, PMID: 33684494

[20] Tigges M Drager S Piccini I Bieber K Vorobyev A Edelkamp J . Pemphigoid disease model systems for clinical translation. Front Immunol. (2025) 16:1537428. doi: 10.3389/fimmu.2025.1537428, PMID: 40165962 PMC11955494

[21] van Delft MAM Aleyd E van der Mast R de Jong N Boon L Simons PJ . Antagonizing FcalphaR1 (CD89) as treatment in IgA-mediated chronic inflammation and autoimmunity. Front Immunol. (2023) 14:1118539. doi: 10.3389/fimmu.2023.1118539, PMID: 37081893 PMC10111428

[22] Shirley M . Stapokibart: first approval. Drugs. (2025) 85:715–20. doi: 10.1007/s40265-025-02151-7, PMID: 40095376

[23] Zhao M Xu Z Wu L Wei Z Yang B Lin Z . Safety and efficacy of stapokibart, an anti-IL-4Rα monoclonal antibody, in children aged 6–11 years with moderate-to-severe atopic dermatitis: An open-label, single-arm phase 1b/2a trial. Br J Dermatol. (2025). doi: 10.1093/bjd/ljaf455, PMID: 41284735

[24] Zhao Y Li JY Yang B Ding YF Wu LM Zhang LT . Long-term efficacy and safety of stapokibart in adults with moderate-to-severe atopic dermatitis: an open-label extension, nonrandomized clinical trial. BioDrugs. (2024) 38:681–9. doi: 10.1007/s40259-024-00668-z, PMID: 39080181

[25] Zhang Y Yan B Zhu Z Wang X Song X Zhu D . Efficacy and safety of stapokibart (CM310) in uncontrolled seasonal allergic rhinitis (MERAK): an investigator-initiated, placebo-controlled, randomised, double-blind, phase 2 trial. EClinicalMedicine. (2024) 69:102467. doi: 10.1016/j.eclinm.2024.102467, PMID: 38356731 PMC10864214

